# A highly flexible and sensitive piezoresistive sensor based on MXene with greatly changed interlayer distances

**DOI:** 10.1038/s41467-017-01136-9

**Published:** 2017-10-31

**Authors:** Yanan Ma, Nishuang Liu, Luying Li, Xiaokang Hu, Zhengguang Zou, Jianbo Wang, Shijun Luo, Yihua Gao

**Affiliations:** 1 Center for Nanoscale Characterization & Devices (CNCD), Wuhan National Laboratory for Optoelectronics (WNLO), School of Physics, Huazhong University of Science and Technology (HUST), Luoyu Road 1037, Wuhan, 430074 China; 2 College of Physics Science & Technology, Guangxi University, Daxue Road 100, Nanning, 530004 China; 3 School of Material Science & Engineering, Guangxi Nonferrous Metals Mineral and Materials, Collaborative Innovation Center, Guilin University of Technology, Jian’gan Road 12, Guilin, 541004 Guangxi China; 4 School of Physics and Technology, Center for Electron Microscopy and MOE Key Laboratory of Artificial Micro- and Nano-Structures, and Institute for Advanced Studies, Wuhan University, Wuhan, 430072 China; 5 School of Sciences, Hubei University of Automotive Technology, Shiyan, 442002 China; 6 School of Material Science and Engineering, Wuhan Institute of Technology, Wuhan, 430205 China

## Abstract

Since the successful synthesis of the first MXenes, application developments of this new family of two-dimensional materials on energy storage, electromagnetic interference shielding, transparent conductive electrodes and field-effect transistors, and other applications have been widely reported. However, no one has found or used the basic characteristics of greatly changed interlayer distances of MXene under an external pressure for a real application. Here we report a highly flexible and sensitive piezoresistive sensor based on this essential characteristics. An in situ transmission electron microscopy study directly illustrates the characteristics of greatly changed interlayer distances under an external pressure, supplying the basic working mechanism for the piezoresistive sensor. The resultant device also shows high sensitivity (Gauge Factor ~ 180.1), fast response (<30 ms) and extraordinarily reversible compressibility. The MXene-based piezoresistive sensor can detect human being’s subtle bending-release activities and other weak pressure.

## Abstract (web summary)

MXenes are a family of layered materials which show promise for a variety of (opto-)electronic applications. Here, the authors leverage the variations of the interlayer distance in Ti_3_C_2_ under external pressure to devise a piezoresistive sensor.

## Introduction

MXenes, as a newly two-dimensional (2D) early transition metal carbides and carbonitrides, were first reported in 2011 by selectively etching out the element “A” from the three-dimensional (3D) structure consisting of MAX^1^. The chemical formula of MXenes is M_*n*+1_X_*n*_T_*x*_, where M represents an early transition metal, X represents carbon and/or nitrogen, *n* = 1, 2 or 3, and T represents a surface termination, such as O, OH and/or F^2, 3^. Similar to graphene, these 2D laminated nanocrystals exhibit large specific surface area, high electrical conductivity, favorable strength and other excellent characteristics^3^. Based on the characteristics above, some important applications were developed. Owing to the surface terminations of function groups (OH, O, F, H, etc.), MXenes have good hydrophilicity for Pb(II) purification and water desalination^4, 5^. Because of the active sites on surface, MXenes can be used as active substrate for catalyst and polymers^6, 7^. Owing to its large specific surface area, good electrical conductivity and convenience of penetration and escape of ions, electrochemical energy storage devices^8–11^, e.g., lithium ion batteries (LIBs) and supercapacitor (SC) were fabricated based on MXenes. However, another essential characteristic of the MXenes is ignored and hence no related applications are exploited. The distance between the neighboring interlayers in MXene can be easily controlled by compression because of its relatively wider interlayer distances fabricated by etching out the element “A” from the MAX^1^. Naturally, the change of the interlayer distances under an external pressure requires a confirmation and hence pressure sensor based on MXenes can be fabricated.

So far, three main types of pressure sensors are recognized, i.e., piezoelectric, capacitive and piezoresistive sensors^12^. Piezoresistive sensors, transducing an external pressure into a resistance signal, have drawn significant attention due to their low-cost fabrication, easy signal collection and number of important applications, such as smart display^13^, skin-inspired electronic devices^14, 15^ and portable healthcare monitors^16, 17^, etc. Compared with the conventional piezoresistive sensor based on brittle metals or the sensing materials fabricated on rigid substrates, the next-generation piezoresistive sensors for wearable healthcare monitoring should not only have high sensitivity, but also high flexibility, compressibility, stretchability and bending properties. To design such a piezoresistive sensor, sensitive sensing materials with suitable geometric electrodes should be fabricated to have a synergistic effect^12^.

In general, the carbonaceous materials, i.e., carbon nanotubes (CNTs), graphene and their composites are suitable materials^16–23^. They have been widely used in flexible and stretchable piezoresistive sensors for high sensitivities by using the deformation of their macrostructures. In this research, CNTs and graphene were fabricated as thin film, smart thread and elastomer foam composite via spraying, vacuum infiltration, and printing and dipping methods^18–23^. However, most of these techniques suffer from a complicated process and/or a delicate design. Nevertheless, the simple coating method leads to the instability of the sensor, because the active sensing materials usually fall out under an outside force. Especially, the inner atomic structure of graphene and CNTs is difficult to be used to further improve the sensitivity of the corresponding sensor for their very high modules (1TPa)^24^ resisting the atomic movement in them. These shortcomings have motivated scientists to find new materials or structures with an easy interlayer atomic movement to reduce costs, simplify the steps and especially importantly improve the sensitivity in order to satisfy the reality requirements. MXenes may have such characteristics due to relatively wide interlayer distances and hence leading to a high sensitivity and flexibility under an external pressure, meeting the requirements of detecting the subtle human’s activities, e.g., eye blinking and cheek bulging.

Until now, a series of MXenes including Ti_3_C_2_, Ti_2_C, Nb_2_C, V_2_C, (Ti_0.5_Nb_0.5_)_2_C, (V_0.5_Cr_0.5_)_3_C_2_, Ti_3_CN and Ta_4_C_3_ have been fabricated^3^. Ti_3_C_2_, as one representative of more than a dozen 2D MXenes, is proved to be simpler and more stable in fabrication technique than other reported MXenes, and have shown broad applications ranging from energy storage devices LIB^8^ and SC^9, 25^, transparent conductive electrodes^26^, electromagnetic interference shielding^27^, field-effect transistor for probing neural activity^28^ based on the good conductivity and to the controllable resistance by the changeable distance between the interlayers in the Ti_3_C_2_ MXene via the intercalation and deintercalation of ions (K^+^, Na^+^, Mg^2+^, etc.)^29–32^. The characteristics of conductivity varied with the changeable distance between the interlayers make Ti_3_C_2_ as a promise material for piezoresistive sensors controlled by the interlayer distance of the lamellar structure under an external pressure, instead of intercalation and deintercalation of ions.

In this study, we fabricated a flexible piezoelectric sensor based on Ti_3_C_2_–MXene with interdigital electrodes, which showed high compressibility, fast response and high sensitivity. An in situ transmission electron microscopy (TEM) study directly illustrated the greatly changed interlayer distances under pressure, supplying the basic working mechanism for the piezoresistive sensor. The MXene-based compress sensor can respond to a wide range of compression with a high Gauge Factor (GF ~ 180.1). The sensor also shows high mechanical reversibility (over 4,000 times) and fast response (<30 ms). We used the MXene-based sensor to monitor and discriminate subtle human activities such as swallowing, coughing, joint bending and so on.

## Results

### Fabrication, Characterization and Sensing Mechanism

Ti_3_C_2_ MXene was prepared by selective etching the Al layer in the precursor Ti_3_AlC_2_ (Supplementary Fig. 1a) with 50% hydrofluoric acid (HF). The scanning electron microscopy (SEM) image confirmed the multilayer structure of resultant (Supplementary Fig. 1b) and the X-ray diffraction (XRD) pattern shows that majority of the Ti_3_AlC_2_ has successfully transformed to Ti_3_C_2_T_X_ (Supplementary Fig. 1c). The surface terminated on Ti_3_C_2_ mainly contained O and F, as shown in the mapping of Ti_3_C_2_T_X_ (Supplementary Fig. 2). Compared with the single Ti_3_C_2_ layer obtained by ultrasonication exfoliation, the multilayered Ti_3_C_2_ MXene has better stability^30^ that the powder of this structure can be stored in ambient condition. This multilayered MXene Ti_3_C_2_ was used as the sensing material in the piezoresistive sensor (Supplementary Fig. 3a) and the fabricating process are shown in the schematic diagram (Supplementary Fig. 4) and illustrated in the Methods.

Briefly, MXene-based sensor consists of three layers, the interdigital electrodes as the bottom layer, the MXene layer as the middle one and the fabric as the top one. The metal interdigital electrodes on the flexible polyimide (PI) film, fabricated by initially ink printing interdigital structure and then magnetron sputtering metal, supply a soft substrate and conductive paths. The distance between two neighboring interdigital electrodes is about 380 ± 20 μm (Supplementary Fig. 5). In comparison with photolithography and other fabrication techniques for interdigital electrode, our method is simple, convenient and non-toxic. Afterwards, the MXene layer was coated on the soft PI substrate by dipping and drying. We optimized the concentration of the MXene solution to ensure the film’s quality, where 250 mg MXene dissolved into 1 ml absolute ethyl alcohol. Subsequently, the fabric with hierarchical structures (Supplementary Fig. 5), as a protective layer, was coated on the MXene layer to fix it under the external mechanical stress. To further improve the stability of the sensor, the polydimethylsiloxane (PDMS) was used to encapsulate the whole device. It is worth noting that the cost of fabrication of MXene-based was low, a drop of MXene solution (30 μl) is enough for the whole sensor. In addition, the soft interdigital electrodes make the sensor flexible and stable under constant bending and compressing.

**Fig. 1:**
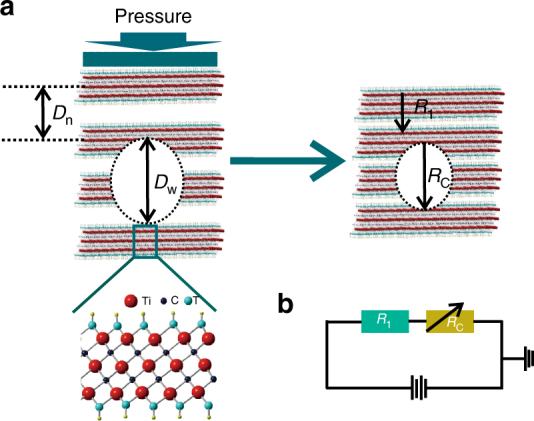
Working micromechanism of MXene-material for piezoresistive sensor. **a** The distances between two neighboring interlayers of the MXenes in the sensor decrease under an external pressure. The wider distance (*D*_w_) between two interlayers is easier to be compressed, while the narrower distance (*D*_n_) between two lattices has a smaller compress ratio. **b** The equivalent circuit diagram of MXene-based piezoresistive sensor, where the total resistance falls due to the distance decrease

Figure 1 shows the working micromechanism of the sensor, where the distance between two neighboring interlayers in MXene (Fig. 1a) will be decreased under an external pressure, in turn reducing the internal resistance *R*_C_ (corresponding to the interlayers with an initial bigger distance) and increasing conductivity, as demonstrated by Fig. 1b and equation *R*_Total_ = *R*_1_ + *R*_C_, where *R*_Total_ is the total resistance, *R*_1_ is another MXene’s part resistivity (corresponding to the interlayers with an initial smaller distance), being a nearly unchanged resistivity under pressure. An external pressure can be monitored via the resistivity changes originated from the changed interlayer distance.

### In situ TEM study on the sensor’s micro dynamic process

**Fig. 2:**
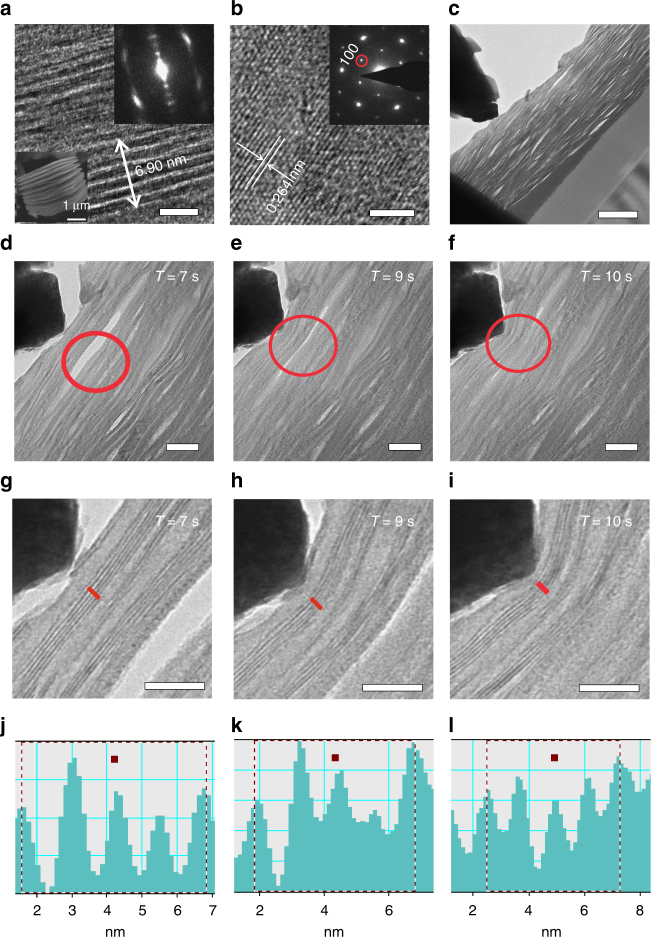
The MXene’s typical microstructure and the in situ dynamic process under an external pressure. **a** Cross-sectional TEM image of the MXene and its two insets shows the lamellar structure. **b** The MXene’s plan view HRTEM image and its corresponding diffraction pattern in the inset, showing well the hexagonal characteristics. **c** In an in situ force instrument, a nanoindenter supported by a tapered needle point is locally applied on the FIB processed MXene sample. **d**–**f** The wider distance in the MXene suffers a rapidly decrease under the external force, from ~12 nm, via ~3 nm to ~0 nm at 7, 9 and 10 s in sequence. **g**–**i** The narrower distance (4 spacings) labeled by a red line ranges from 5.23 nm, via 4.98 nm then to 4.81 nm at 7, 9 and 10 s in sequence. **j**–**l** The analysis on **g**–**i** using the digital micrograph software. Measured from the original state in **g**, the strain values were 4.78 and 8.03% at the states **h**, **i**, respectively. Scale bar for **a**, **b** is 4 nm, **c** is 200 nm, **d**–**f** is 40 nm and **g**–**i** is 20 nm

Figure 2 shows the MXene’s typical microstructure and the in situ dynamic process under an external pressure. The TEM images of Fig. 2a and its two insets display the obvious lamellar structure and the average interlayer distance of 1.15 nm. Figure 2b shows the MXene’s plan view high-resolution TEM image and its corresponding diffraction pattern, where the hexagonal characteristics can be recognized well with the (100) plane distance of 0.264 nm. As shown in Fig. 2c, a lot of bright linear empty regions existed. A statistical analysis on the width are made for 177 empty regions and shown in Supplementary Fig. 3b. Majority of them have a wide interlayer distance of 3–12 nm, corresponding to an emptiness of 3–12 ordinary average interlayers. As also shown in Fig. 2c, a nanoindenter^33, 34^ supported by a tapered needle point (an in situ force instrument) is locally applied on the focused ion beam (FIB) processed MXene sample. The wide interlayer distance circled in red in Fig. 2d–f decreases rapidly under the external force. With the increase of the compressive force, the wide distance ~12 nm sharply decreases until it is near to ~3 and ~0 nm with compress ratios 75% at 9 s and 100% at 10 s. Meanwhile, the ordinary distance between the MXene interlayers also gradually reduced with the increase of the external force, but much smaller than that of the rate of the wider distance. For example, the shorter distance of 4 layers labeled by a red line ranges from 5.23 nm, via 4.98 nm then to 4.81 nm at 7, 9 and 10 s in sequence, as shown in Fig. 2g–i (the analysis process in Fig. 2j–l using the digital micrograph software). Measured from the original state in Fig. 2g, the strain ratios were 4.78% in Fig. 2h and 8.03% in Fig. 2i. Moreover, when the pressure was removed quickly, both the wider and ordinary distances between the interlayers can return back quickly, indicating the reversibility, as shown in Supplementary Movies 1 and 2. In brief, the in situ TEM study shows that the distances between the interlayers of MXene can be reduced and reversibly recovered by an external force on/off, respectively, as shown in Supplementary Fig. 6. Furthermore, the wider distance has a higher compress rate than that of the narrow distance.

### The properties of the piezoresistive sensor

**Fig. 3:**
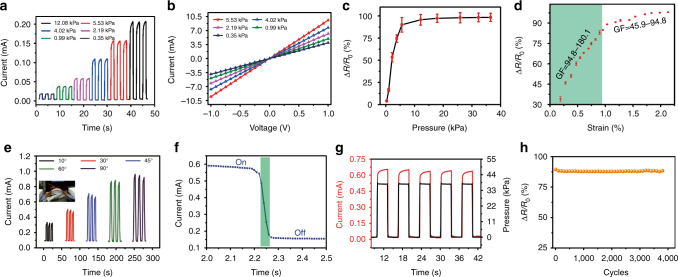
The response of the MXene-based sensor to pressures. **a** The I–T curves under the pressures below 13 kPa. The current monotonically increases with the pressure. **b** The linear relation of the I–V curves suggests the ohmic contacts between MXene and interdigital electrodes. **c** The *∆R/R*_off_ first undergoes a sharp increase as function of pressure below 5 kPa, then a small increase above 5 kPa. Here, the SD for the data is in the range of 1.44–8.21%, which is shown by the error bars and calculated by analyzing five sets of data under each pressure. **d** The *∆R/R*_off_ vs strain. The highest GF, 180.1 was obtained. Here, the SD for the data is in the range of 0.26–1.78%, which is shown by the error bars and calculated by analyzing four sets of data under each strain value. **e** I–T relation under various bending angles. **f** An amplifying I–T curve part exhibiting a fast responsive time <30 ms. **g** Both the output current and external pressure on time kept well in step with the loading and unloading. **h** The resistivity change ratio *∆R/R*_off_ keeps a good stability

To study the piezoresistive effect of MXene strain sensors, we performed the compressive measurement under various levels of pressure (see in the experimental section). Figure 3a, showing the I–T curves with the pressure below 13 kPa, demonstrates that the electric current monotonically increases with the pressure value. Each curve is stable and continuous without obvious signal attenuation under each loading and unloading (shown in Supplementary Fig. 3d). Our sensor can clearly detect a very small pressure 351 Pa. Furthermore, the linear relation of the I–V curves (Fig. 3b) from −1 to 1 V suitable for sensors suggests that ohmic contacts were formed between MXene and interdigital electrodes. With the increase of the pressure, the slope of I–V curves increased, indicating the continuous decrease of the sensor’s resistivity. We measured the *∆R/R*_off_ with respect to the pressure value as shown Fig. 3c. The *∆R/R*_off_ first underwent a sharp increase below 5 kPa, then a small increase above 5 kPa. It is speculated that the major resistance change under 5 kPa is ascribed to the interlayers between the MXene layers, which correspond to the initial compress Fig. 2d–i. In addition, the contact between MXene bulk is not very close. When imposing on large pressure above 5 kPa it becomes tight, leading to further increase of resistance change. But the variation is small. The GF (GF* = ΔR/R*_off_ */ε*), i.e., the sensitivity of the MXene film to strain was shown in Fig. 3d. The GF ratios are 180.1–94.8 and 94.8–45.9 in the ranges of 0.19–0.82% and 0.82–2.13% strain, respectively, higher than carbon materials, metal nanowires and MoS_2_, etc., as shown in Supplementary Table 1. In the very small strain range of 0.19–0.82%, the sensitivity of the MXene-based sensor was extremely high and then suffered a sharp decrease above 0.82%, finally gradually saturated when the strain was over 1.80%. By modifying the reaction conditions, partial-exfoliated MXene was obtained, and most of layers didn’t separate completely^35^. The sensor fabricated by the partial-exfoliated MXene tended to exhibit a lower sensitivity than that of well layered MXene (Supplementary Fig. 7). Even under a large scale pressure, the Δ*R/R*_off_ of the sensor based on partial-exfoliated MXene also showed no significant change. It is obvious that the quality of MXene is a key to the sensitivity of the sensor.

The responsive resistivity varied at two pressure regions, making it capable of detecting different degree human movements, such as joint bending and breathing. The interdigital electrodes fabricated on a soft PI film naturally own flexibility, endowing our sensor with bending and torsion properties. In order to study the strain response properties of the MXene-based strain sensor at various angles, we investigated the current output by mounting the as-synthesized strain sensor on a bending machine. On the machine, the MXene-based process also exhibited good recovery performance, quickly returning to the initial state without degradation after the bending. In Fig. 3e, a series of I-T curves under various bending angles were measured. The resistivity change was proportional to the bending angles within a range below 70°. Although the bending angle exceeds 70°, the resistivity change of the MXene-based sensor tended to saturation, which is similar to the situation under various pressures in Fig. 3c. The MXene-based sensor is connected with a light-emitting diode (LED) light to form a series circuit under a power supply of 2 V, to vividly demonstrate the high sensitivity under an external force, as illustrated in Supplementary Fig. 8a. Figure 3f shows an amplifying I–T curve of our based-MXene sensor, exhibiting a fast response time <30 ms. The fast response (without hysteresis) ensures the sensor sensing in time under an external force. Moreover, both the output current and external pressure kept well in step with the loading and unloading, as shown in Fig. 3g. To evaluate the mechanical durability of the MXene-based strain sensor, the loading-unloading test under 7.5 kPa was performed. After 4,000 cycles, the sensor signal showed little attenuation, as shown in Fig. 3h. During the first 100 cycles, the MXene-based sensor suffered a minor attenuation (Supplementary Fig. 8b), which is normal for sensors^17, 36, 37^. In addition, there were no obvious defects or delamination after more than 100 loading–unloading cycles, which was proved by the in situ SEM imaging of multilayer MXene (Supplementary Fig. 9 and Supplementary Movies 3). The excellent stability of the MXene-based sensor shows a great potential for applications. Figure 3i quantitatively evaluates the *∆R/R*_off_ change during the repeated compressive cycles. The correlation curves were basically consistent with each other below 7.5 kPa after more than 100 times loading (red)/unloading (blue) (Supplementary Fig. 8c).

### The applications of the sensor for human’s activities etc

**Fig. 4:**
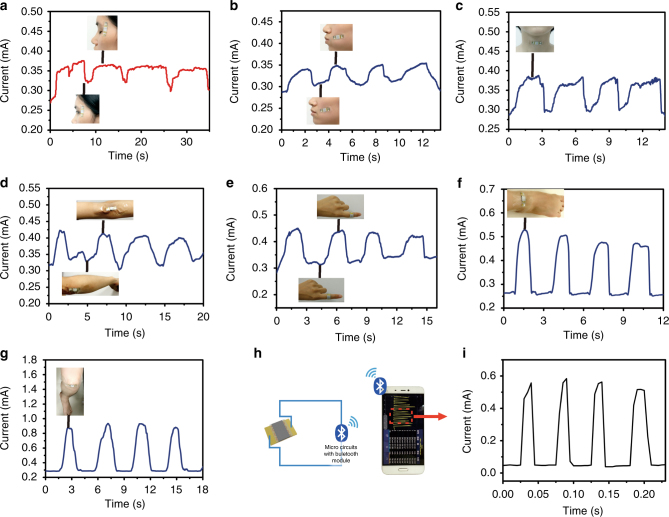
The MXene-based sensor was used to explore the full-range human activities. The current change of the sensor, corresponding to minimal strain change of eye blinking **a** cheek bulging **b** and throat swallowing **c** were precisely recorded. **d**–**f** The current change for the bending-release movement of the elbow **d**, fingers **e** and ankle **f** were also recorded. **g** The I–T curve of knee bending-release. **h** The sensor was connected in series to a micro circuit implanted with a Bluetooth, which transformed the current signal to a mobile phone. **i** The knee motion from the micro circuit implanted with a Bluetooth is quite similar to that from the Agilent equipment both in amplitude and shape, but in a faster frequency than that in **g**

The MXene-based sensor possesses good flexibility, high sensitivity and a wide range of strain gauge, so it was used to explore the potential applications for full-range recognition of human activities. We directly attached it on the muscle or joint and then sealed with the transparent tape, e.g., outside of an eye corner (insets in Fig. 4a), the cheek (insets in Fig. 4b) and the throat (inset in Fig. 4c), to monitor the subtle motion resulting from microexpression. The current change of the sensor, corresponding to minimal strain change of eye blinking, cheek bulging and throat swallowing, were precisely recorded, as shown in Fig. 4a–c, respectively. The relative I–T curves are different and distinguishable by comparing the shape and intensity change of the plot. Besides, the bending-release movement of the elbow, fingers and ankle in Fig. 4d–f are also recorded. The current change (Δ*I/I*_off_ *)* of the knee bending-release movements is the highest among the human activities. Moreover, the I–T curves of knee bending-release are shown in Fig. 4g. The above results demonstrate that the sensor switched rapidly at loading and unloading, where the current value remained nearly identified under the same motion. Thus, the MXene-based sensor can perform accurate detection, due to its good flexibility and high sensitivity. For portable application, the sensor was connected in series to a micro circuit implanted with a Bluetooth system, which transformed the varied current or resistance changes into wireless electromagnetic wave signals, as illustrated in Fig. 4h, i. Here, the conductivity of MXene is about 6,500 S cm^−1^, which is higher than graphene and CNTs^9, 26^. The output current usually exceeds the reported sensor and the noise impact from outside is minimal. It is obvious that the portable MXene-based sensor with a micro circuit Bluetooth system can also response the knee motion quickly.

**Fig. 5:**
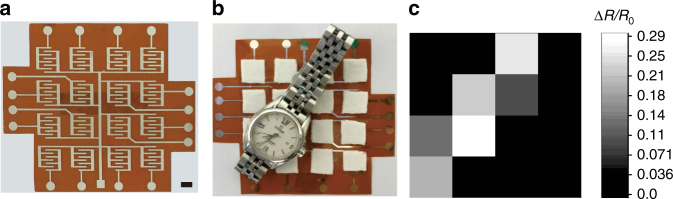
The as-fabricated 4 × 4 MXene-based sensor arrays. **a** 4 × 4 MXene-based sensor pixel arrays to detect pressure distribution (scale bar is 5 mm). **b** A watch was placed on the sensor arrays. **c** The corresponding output at each pixel was recorded and measured. The color contrast in **c** mapping local pressure distribution agreed with the watch position in **b**

We also design a 4 × 4 MXene-based sensor pixel array to detect the pressure distribution by ink-jet printing and magnetron sputtering, as shown in Fig. 5a, b. Each sensing pixel was connected with flexible Ag electrodes based on PI film with a resistance low to dozens of ohms. In comparison with the whole system, the resistance valve can be limited in the measurement procsess, thus each pixel did not interfere with each other. When a watch was placed on the surface of the sensor arrays, the corresponding output at each pixel was recorded and measured, as shown in Fig. 5c, where the color contrast mapping local pressure distribution agreed with the watch position in Fig. 5b. Consequentially, the portable and flexible piezoresistive sensor based on MXene Ti_3_C_2_T_X_ shows potential application in wearable devices and electronic skin, which opens up a new field for other MXenes.

## Discussion

A highly flexible and sensitive MXene-based piezoresistive sensor was fabricated on the basic characteristics of greatly changed interlayer distances under an external pressure. An in situ TEM study directly illustrated the basic working mechanism for the piezoresistive sensor. The MXene-based sensor displayed fast response, good stability and a wide application in detecting subtle human activities and other weak pressure. The GF value reached to 180.1, which is significantly higher than that of other reports. To the best of our knowledge, it is the first piezoresistive sensor to utilize the MXene (Ti_3_C_2_) with the essential characteristic and opens up a new field for the application of other MXenes.

## Methods

### Chemicals

The MAX (Ti_3_AlC_2_) powder and HF were purchased from Beijing Wisdom Company and Aladdin Reagent Company, respectively. Milli-Q water (18.2 MΩ, resistivity) was used for all solution preparations.

### Synthesis of multilayer MXene

The commercial MAX powder was firstly grinded and then heated at 1350 °C for 2 h in argon atmosphere to further improve the quality of the precursor. After that, the HF (50% wt) was slowly added into the pre-processed powder. Then the mixture reacted for 24 h under stirred at 50 °C. After that, the resulted solution was centrifuged for several times until the pH reached to about 6. The final sediment was then dried for 12 h at the vacuum oven.

### Fabrication of the interdigital electrodes

A PI film substrate was firstly tailored into proper size. Then, the PI film was cleaned by acetone, ethanol and deionized water for several times. Immediately following the rinsing thoroughly, the interdigital patterns were printed on the PI film by commercial ink-jet printer (HP Deskjet 1010). After that, metal Au or Ag was respectively deposited on it by Megnetron Sputtering. At last, the printed circuit template was lifted off by sonication in ethanol or deionized water.

### Preparation of the sensor based on MXene

A PI film substrate was firstly tailored into proper size. Then, the PI film was cleaned by acetone, ethanol and deionized water for several times. Immediately following the rinsing thoroughly, the interdigital patterns were printed on the PI film by commercial ink-jet printer (HP Deskjet 1010). After that, metal Au and Ag was respectively deposited on it by Megnetron Sputtering. At last, the printed circuit template was lifted-off by sonication in ethanol or deionized water. The MXene-based sensor was prepared by sandwiching the Ti_3_C_2_ layer between the flexible interdigital electrode and fabric. In brief, a drop of solution containing MXene obtained by mixing the above dried Ti_3_C_2_ powder with ethanol solution was dropped onto the middle of the interdigital electrode. After drying, a piece of cotton cloth with a slightly larger area than that of Ti_3_C_2_ was placed as the top layer to prevent falling out from the substrate. The both ends of the flexible interdigital electrodes were sealed during dropping MXene until it becomes dry. Finally, the ends of PI film were connected with two copper strips by silver paste.

### In situ sample preparation and experimental methods

The in situ TEM sample was fabricated by using a dual-beam SEM/FIB microscope (FEI Quanta 3D FEG) equipped with nanomanipulator (Oxford Instruments OmniProbe 100) and gas injection system. During the in situ TEM (JEOL JEM-2010 FEF) study process, a blunt tungsten tip driven by Nanofactory system (EP1000) was employed to impose the external force. The MXene layers were imaged in real time using diffraction contrast imaging (diffraction **g** = (200)) and recorded by a charge-coupled device (Gatan) in JEOL 2010F. The distance change between MXene layers under pressure is evaluated by using the digital micrograph software. Therefore, the strain value (defined as Δ*l/l*) can be calculated.

### Characterizations and Measurements

The morphology and thickness of MAX, MXene and MXene-based sensor were investigated by high-resolution field-emission SEM (FEI Nova NanoSEM 450, 10 kV) and TEM (FEI Titan G260–300). XRD of MAX and MXene were carried out using a Rigaku X-ray diffractometer with Cu *Kα*-radiation and Ni filter. To test the response of our pressure sensors under static and dynamic forces, a system with a computer controlled stepping motor, a force sensor and an electrochemical workstation (Aglient B2901A) were used. The input voltage was set as 1 V during the tests.

### Data availability

The authors declare that the data supporting this study are available within the article. Furthermore, extra data are also available from the corresponding author upon request.

## Electronic supplementary material

Supplementary Information

Description of Additional Supplementary Files

Supplementary Movie 1

Supplementary Movie 2

Supplementary Movie 3
