# Supplementary material for: A highly flexible and sensitive piezoresistive sensor based on MXene with greatly changed interlayer distances
Source: Nat Commun. 2017 Oct 31;8:1207. doi: 10.1038/s41467-017-01136-9 (PMC5663936; doi:10.1038/s41467-017-01136-9)
Supplement: Supplementary file 1 — Supplementary Information [file 41467_2017_1136_MOESM1_ESM.pdf]

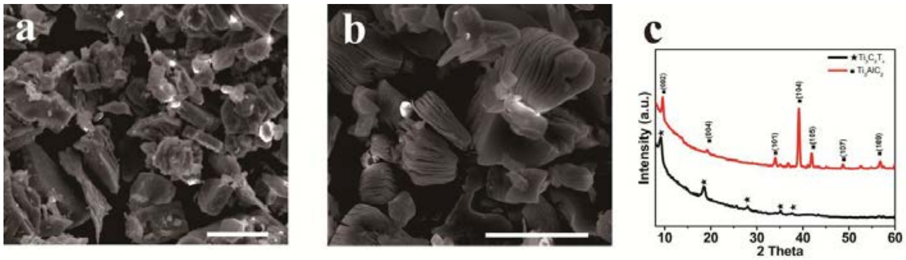

Supplementary Figure 1 | Microstructure of MAX (Ti_3AlC_2) and MXene (Ti_3C_2). (**a**), (**b**) Representative SEM images of MAX (Ti_3AlC_2) and MXene (Ti_3C_2). Scale bars, 5 µm. (**c**) XRD patterns of MAX (Ti_3AlC_2) and MXene (Ti_3C_2).

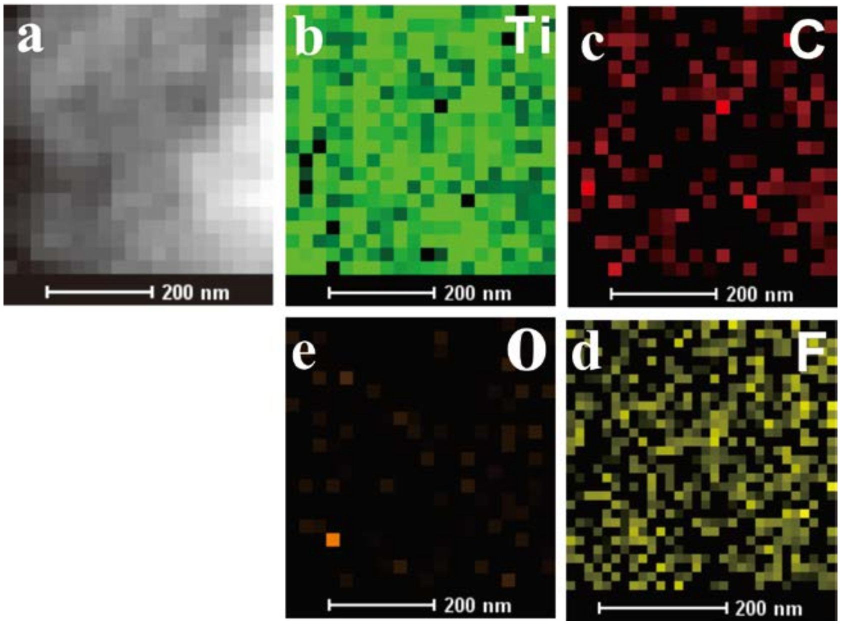

Supplementary Figure 2 | Plan-view of a MXene sheet and its elements' mapping. (**a**) A sheet of MXene. (**b-f**) The Ti, C, F and O element mapping of the MXene.

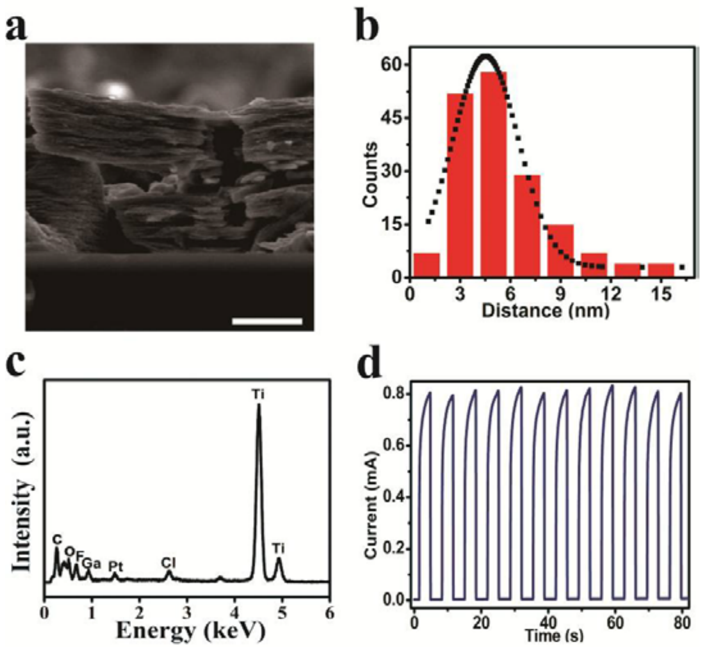

Supplementary Figure 3 | (**a**) The cross-section SEM image of the MXene on PI film. (**b**) A statistical analysis on the width for 177 empty regions. Scale bar, 1 μm. (**c**) The x-ray energy dispersion spectrum (EDS), showing the existence of the Ga element, which should be from the empty regions with a width <3 nm covered by a thin Ga layer during focus ion beam (FIB) milling of Ga-ions. (**d**) The response is stable and continuous without obvious signal attenuation under each loading and unloading.

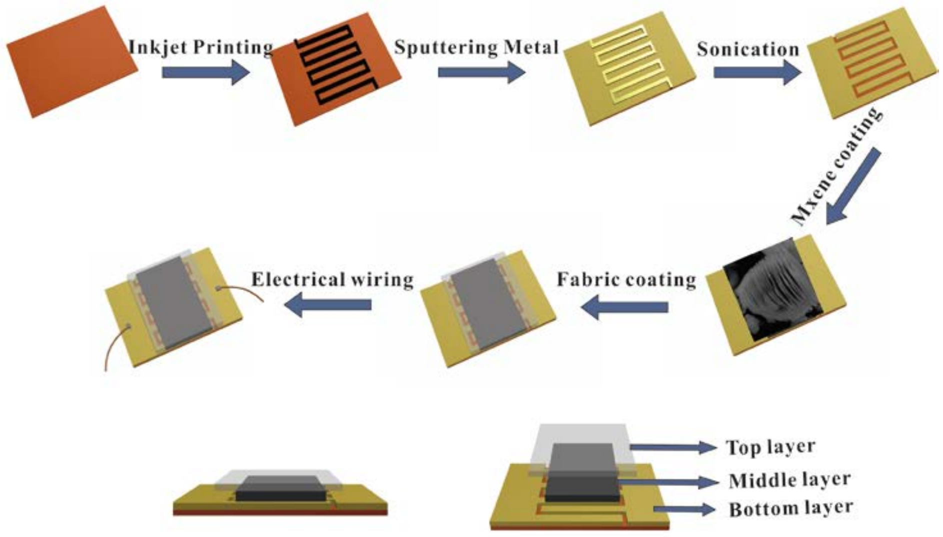

Supplementary Figure 4 | Preparation diagram of the MXene-based sensor.

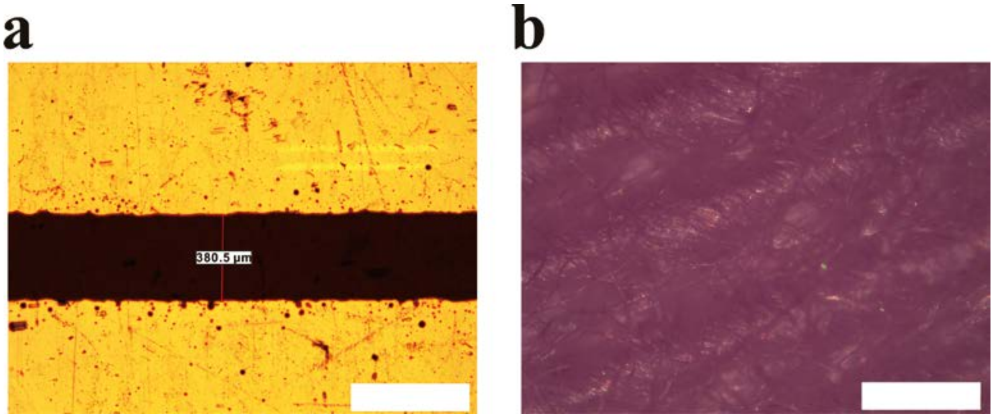

Supplementary Figure 5 | Optical image of the interdigital electrodes (**a**) **and the fabric** (**b**)**.** Scale bars, 500 µm.

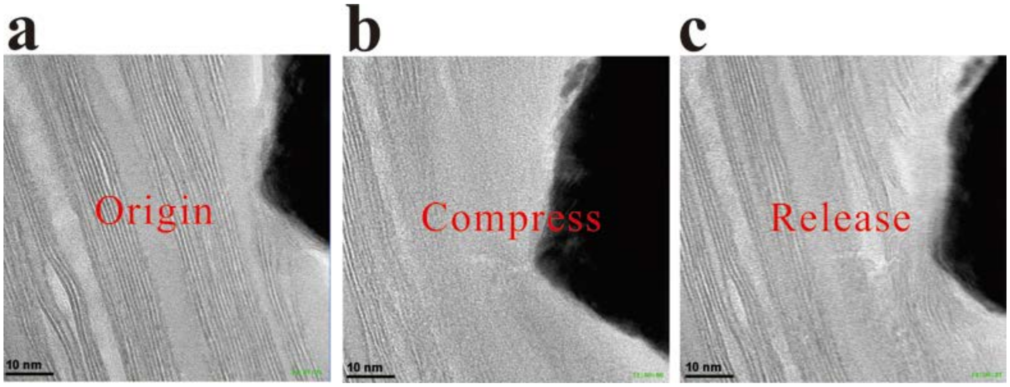

Supplementary Figure 6 | The compress and release process of MXene. (a) The original state. **(b)** The compress state. **(c)** The release state.

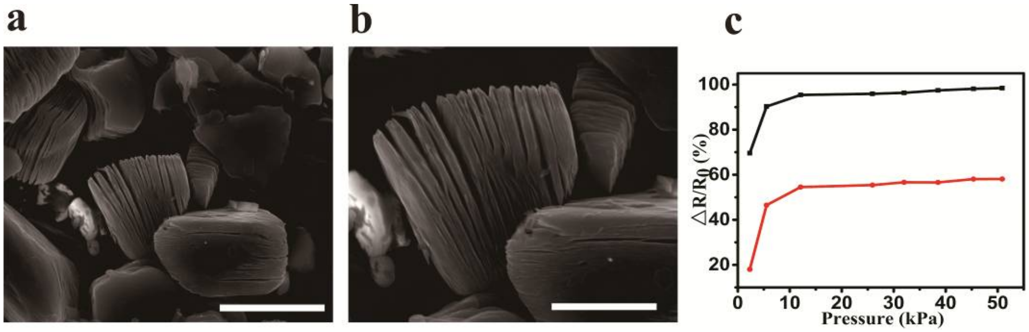

Supplementary Figure 7 | The sensor fabricated by partial-exfoliated MXene and the representative SEM images (**a**)**,** (**b**). (**c**) The MXene tends to exhibit a lower sensitivity (red curve) than that (black curve) of a well layered MXene**.** Scale bars for **a** and **b** is 2 µm and 1µm, respectively.

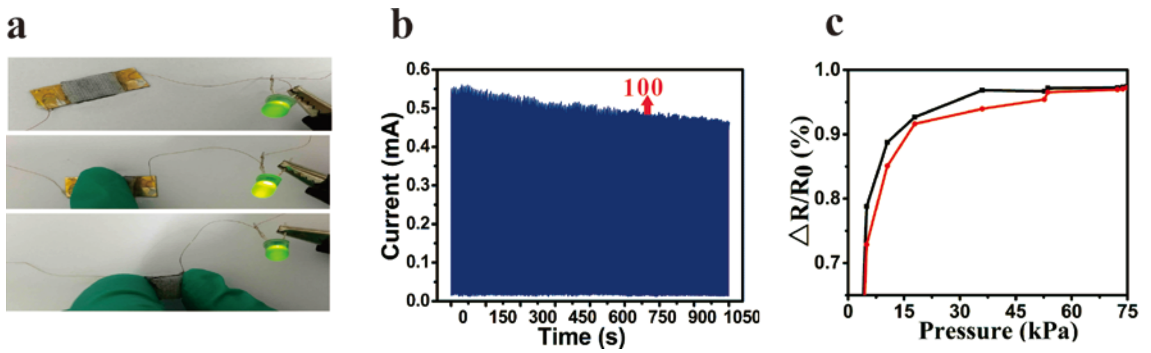

Supplementary Figure 8 | The performance of the MXene-based sensor. (**a**) The sensor is connected with a LED bulb to form a series circuit under a power supply of 2V to vividly demonstrate the high sensitivity under an external force. (**b**) During the first 100 cycles, the performance of the MXene-based sensor suffered a minor attenuation. (**c**) The cyclic compressive tests were quantitatively conducted after more than 100 times loading (red)/unloading (black).

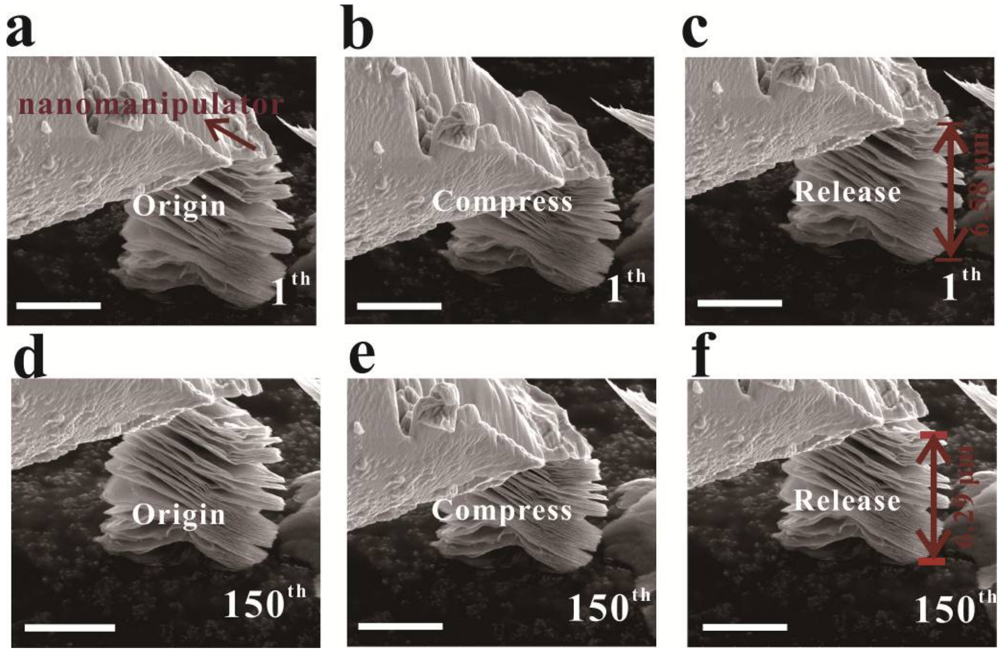

Supplementary Figure 9 | *In-situ* SEM images comparisons of multilayer MXene under compress of a nanomanipulator. (**a**), (**d**) the images of the origin state of MXene; (**b**), (**e**) the images of the compressed state of MXene; (**c**), (**f**) the images of the released state of MXene. **a, b & c** displayed first loading-unloading cycle, **d, e & f** displayed the 150th loading-unloading cycle. Scale bars, 4 μm.

Supplementary Table 1 | The characteristics comparison of the pressure sensor based on various materials.

Materials	Sensing range	Sensitivity	Reference	
Gold Nanowires	5 − 15%	7.38	(*1*)	
Carbonized Silk Fabric	< 250	9.6	(*2*)	
	250–500%	37.5		
Carbon Black@Polyurethane Sponge	< 9%	2.2	(*3*)	
ZnO Nanowires	12.0-6.9 mm (bending radius)	7.6	(*4*)	
Graphene–Nanocellulose	10 − 100%	1.6-7.1	(*5*)	
Percolative graphene film	< 1.7%	15	(*6*)	
CNT Foam Sensors	< 30%	2.63	(*7*)	
CNT/Ag sponges	10 − 90%	6.13	(*8*)	
MoS_2	< 1.98%	74.4	(*9*)	
Our work	0.19−0.82 %	94.8-180.1		
	0.82−2.13 %	45.9-94.8		

Supplementary References

1. Gong, S. *et al*. A wearable and highly sensitive pressure sensor with ultrathin gold nanowires. *Nat. Commun.* **5**, 3132 (2014).
2. Wang, C.Y. *et al*. Carbonized Silk Fabric for Ultrastretchable, Highly Sensitive, and Wearable Strain Sensors. *Adv. Mater.* **28**, 6640-6648 (2016).
3. Wu, X. *et al*. Large-Area Compliant, Low-Cost, and Versatile Pressure-Sensing Platform Based on Microcrack-Designed Carbon Black@Polyurethane Sponge for Human-Machine Interfacing. *Adv. Funct. Mater.* **26**, 6246-6256 (2016).

4. Lee, T. *et al.* Flexible Textile Strain Wireless Sensor Functionalized with Hybrid Carbon Nanomaterials Supported ZnO Nanowires with Controlled Aspect Ratio. *Adv. Funct. Mater.* **26**, 6206-6214 (2016).
5. Yan, C.Y. *et al*. Highly stretchable piezoresistive graphene-nanocellulose nanopaper for strain sensors. *Adv. Mater.* **26**, 2022-2027 (2014).
6. Hempel, M. *et al.* A Novel Class of Strain Gauges Based on Layered Percolative Films of 2D Materials. Nano Lett. 12, 5714-5718 (2012).
7. Li, Y. *et al.* Poisson Ratio and Piezoresistive Sensing: A New Route to High-Performance 3D Flexible and Stretchable Sensors of Multimodal Sensing Capability. *Adv. Funct. Mater.* **26**, 2900-2908 (2016).
8. Zhang, H. *et al.* Piezoresistive Sensor with High Elasticity Based on 3D Hybrid Network of Sponge@CNTs@Ag NPs. *Acs Appl. Mater. Inter.* **8**, 22374-22381 (2016).
9. Park, M. *et al.* MoS_2-Based Tactile Sensor for Electronic Skin Applications. *Adv Mater.* **28**, 2556-2562 (2016).
