# Supplementary material for: A highly flexible and sensitive piezoresistive sensor based on MXene with greatly changed interlayer distances
Source: Nat Commun. 2017 Oct 31;8:1207. doi: 10.1038/s41467-017-01136-9 (PMC5663936; doi:10.1038/s41467-017-01136-9)
Supplement: Supplementary file 2 — Description of Additional Supplementary Files [file 41467_2017_1136_MOESM2_ESM.pdf]

Description of Additional Supplementary Files

File Name: Supplementary Movie 1
Description: A blunt tungsten tip imposed on the multilayer MXene, which offered the external force. It is obviously displayed that the distances between the interlayers of MXene were gradually reduced with the increase of the external force.

File Name: Supplementary Movie 2
Description: The distances between the interlayers of MXene recovered reversibly when the external force was removed.

File Name: Supplementary Movie 3
Description: The in situ SEM observation of the interlayer change of MXene during 100 loading-unloading cycles.
